# Association of serum C‐reactive protein with in vivo cerebral beta‐amyloid deposition in nondemented older adults

**DOI:** 10.1002/alz.088148

**Published:** 2025-01-09

**Authors:** HyeJi Choi, Min Soo Byun, Dahyun Yi, Joon Hyung Jung, Bo Kyung Sohn, Musung Keum, Gijung Jung, Hyejin Ahn, Jun‐Young Lee, Yun‐Sang Lee, Yu Kyeong Kim, Dong Young Lee, the KBASE Research Group

**Affiliations:** ^1^ Department of Neuropsychiatry, Seoul National University Hospital, Seoul Korea, Republic of (South); ^2^ Department of Psychiatry, Seoul National University College of Medicine, Seoul Korea, Republic of (South); ^3^ Institute of Human Behavioral Medicine, Medical Research Center, Seoul National University, Seoul Korea, Republic of (South); ^4^ Department of Psychiatry, Chungbuk National University Hospital, Cheongju Korea, Republic of (South); ^5^ Sanggye Paik Hospital, Inje University College of Medicine, Seoul Korea, Republic of (South); ^6^ Hwajung Hospital, Goyang, Gyeonggi‐do Korea, Republic of (South); ^7^ Interdisciplinary program of cognitive science, Seoul National University, Seoul Korea, Republic of (South); ^8^ Department of Neuropsychiatry, SMG‐SNU Boramae Medical Center, Seoul Korea, Republic of (South); ^9^ Department of Nuclear Medicine, Seoul National University College of Medicine, Seoul Korea, Republic of (South)

## Abstract

**Background:**

C‐reactive protein (CRP) is widely used in clinical practice as a marker for inflammatory condition. Previous studies have examined the association between CRP levels in peripheral blood and Alzheimer’s disease (AD) dementia, but have yielded mixed results. In this study, we investigated the association between serum CRP levels and in vivo cerebral beta‐amyloid (Aβ) deposition in nondemented older adults.

**Method:**

Nondemented older adults were recruited from the Korean Brain Aging Study for the Early Diagnosis and Prediction of Alzheimer's disease (KBASE) cohort. All participants underwent ^11^C‐PiB‐PET/MRI and blood sampling. Serum CRP was measured by using a turbidimetric immunoassay (TIA) method. Since CRP level lower than 3 mg/L is considered as normal range in clinical settings, we classified the participants into two groups as follows: i) CRP < 3mg/L and ii) CRP ≥ 3mg/L. CRP values that deviated more than 3 standard deviations from the mean were considered outliers and excluded from analysis. Global cortical PiB uptake was used for measurement of global Aβ deposition. Both log‐transformed CRP and global PiB SUVR was used for analysis. Multiple linear regression analysis with CRP as an independent variable and global PiB SUVR as a dependent variable was performed adjusting covariates including age, sex, and apolipoprotein E ε4 (APOE4) positivity.

**Result:**

Total 393 nondemented (260 cognitively normal and 133 mild cognitive impairment) older adults were included in the analysis. In CRP <3mg/L groups (N=393), CRP level showed significant negative association with global Aβ deposition, after adjusting age, sex and APOE4 positivity (β[SE] = ‐0.036 [0.015], p=0.018). In contrast, no significant association between peripheral blood CRP level and global Aβ deposition was observed when CRP level was higher than 3mg/L (N=37).

**Conclusion:**

Our finding suggests that CRP in blood inversely relate to Aβ deposition in brain when the levels of CRP are within normal range (i.e., relatively low). However, such relationship appears not persist when CRP levels are higher than normal limit. Further studies are needed to understand the mechanisms underlying the relationship between peripheral CRP and cerebral Aβ accumulation.

